# Human iPSC-Derived Muscle Cells as a New Model for Investigation of EDMD1 Pathogenesis

**DOI:** 10.3390/ijms26041539

**Published:** 2025-02-12

**Authors:** Marta Lisowska, Marta Rowińska, Aleksandra Suszyńska, Claudia Bearzi, Izabela Łaczmańska, Julia Hanusek, Amanda Kunik, Volha Dzianisava, Ryszard Rzepecki, Magdalena Machowska, Katarzyna Piekarowicz

**Affiliations:** 1 Laboratory of Nuclear Proteins, Faculty of Biotechnology, University of Wrocław, 50-383 Wrocław, Poland; 2 Institute for Biomedical Technologies, National Research Council, 20054 Segrate, Milan, Italy; 3 Department of Genetics, Wrocław Medical University, 50-368 Wrocław, Poland

**Keywords:** emerin, *EMD* mutation, Emery–Dreifuss muscular dystrophy, skeletal muscles, stem cells, disease modeling, muscle differentiation in vitro

## Abstract

Emery–Dreifuss muscular dystrophy type 1 (EDMD1) is a rare genetic disease caused by mutations in the *EMD* gene, which encodes the nuclear envelope protein emerin. Despite understanding the genetic basis of the disease, the molecular mechanism underlying muscle and cardiac pathogenesis remains elusive. Progress is restricted by the limited availability of patient-derived samples; therefore, there is an urgent need for human-specific cellular models. In this study, we present the generation and characterization of induced pluripotent stem cell (iPSC) lines derived from EDMD1 patients carrying *EMD* mutations that lead to truncated or absent emerin, together with iPSCs from healthy donor. The patient-specific iPSCs exhibit stable karyotypes, maintain appropriate morphology, express pluripotency markers, and demonstrate the ability to differentiate into three germ layers. To model EDMD1, these iPSCs were differentiated into myogenic progenitors, myoblasts, and multinucleated myotubes, which represent all stages of myogenesis. Each developmental stage was validated by the presence of stage-specific markers, ensuring the accuracy of the model. We present the first iPSC-based in vitro platform that captures the complexity of EDMD1 pathogenesis during myogenesis. This model can significantly contribute to understanding disease mechanisms and develop the targeted therapeutic strategies for EDMD1.

## 1. Introduction

Emery–Dreifuss muscular dystrophy type 1 (EDMD1, OMIM #310300) is a rare genetic disorder with a prevalence of one per 100,000 male births. It is an X-linked recessive disease caused by mutations in the *EMD* gene (*locus Xq28*) coding for nuclear envelope (NE) protein emerin [1]. The main symptoms of EDMD1 are observed in the skeletal muscles and heart, including muscle weakness and wasting, tendon contractures, and cardiac dysfunction [2]. The disease is incurable, with a substantial risk of sudden death in middle age caused by heart block. It is still unclear whether the changes observed in progenitor cells or at the later stages of myogenesis are crucial for aberrations in muscle development and regeneration in EDMD1. Understanding the disease mechanisms is fundamental for design, development, and evaluation of the therapy.

Emerin is a transmembrane protein with a LAP2, emerin, MAN1 (LEM) domain that interacts with various partners in the cell nucleus, including the highly conserved DNA-binding protein BAF (Barrier-to-Autointegration Factor), facilitating DNA tethering to NE. Emerin plays an important role in nuclear stability, gene regulation, chromatin organization, and the cell cycle [3,4,5]. It is especially important in striated muscle cells, where emerin is essential for muscle development, maintenance, and regeneration [6,7,8]. The absence or misexpression of emerin leads to muscle degeneration. Furthermore, emerin, together with another NE protein, lamin A/C, appears critical for muscle cell differentiation [9]. Dysfunction of satellite cells (SCs), the progenitors of muscle cells, has also been hypothesized to contribute significantly to the progression of EDMD by impairing myofiber repair and regeneration [10]. Despite this, the role of emerin in precursor muscle cells remains poorly understood. One major limitation is the lack of appropriate models for studying emerin’s role in pathogenesis. Not enough data have been acquired with human, patient-derived muscle precursor cells, with an investigation of patients-derived SCs mostly restricted to microscopic analysis of rare muscle biopsies [11,12,13].

The function and regulation of emerin are species-dependent, limiting the utility of mouse models and non-human cells [14,15]. While emerin-null mice exhibit only minor phenotypic changes, such as delayed skeletal muscle regeneration and repair, mild atrioventricular alterations, and motor coordination defects, humans with emerin deficiency develop muscular dystrophy. Additionally, the mutation-related phenotype in humans significantly depends on genetic background [16,17,18]. It is possible to preserve it using an approach based on MyoD overexpression in the patient’s fibroblasts to obtain myoblasts [19], but this model does not allow for studying myogenic precursors as well as MyoD-related signaling pathways may be influenced. The absence of accessible human-derived muscle tissue is a significant barrier for investigating EDMD1 in vitro. The availability of patients’ skeletal muscle biopsies is very limited, and especially patients’ cardiomyocytes are not available for research. Therefore, patient-specific induced pluripotent stem cells (iPSCs) offer a promising alternative for providing human muscular in vitro models. Importantly, as the starting material is derived from patients, the model retains the patient-specific genetic background, providing a physiologically relevant system. Differentiated muscle cells derived from iPSCs could serve as a key model for EDMD1, to elucidate disease mechanisms and the development of targeted therapies.

iPSCs can be differentiated into striated muscle progenitors and subsequently into myoblasts and multinucleated myotubes using a transgene-free protocol [20]. This approach enables the generation of a sufficient amount of research material. The initial step of the iPSCs differentiation procedure produces muscle progenitors, also called SCs. However, this method does not allow for obtaining separately quiescent and activated SCs populations. Single-cell transcriptomic analyses of muscle progenitors obtained via iPSCs differentiation using various chemical methods revealed that their expression profiles closely resemble those of embryonic skeletal muscle progenitor cells while also sharing substantial similarity with the adult muscle SCs population [21].

SCs are typically characterized by the expression of the transcription factors belonging to the paired box family—Pax3 and Pax7, which serve as key markers of this cell population. They are both essential regulators of myogenesis. Pax3 is involved in the early stages of muscle development by regulating downstream regulatory factors MyoD and Myf5. Pax7 is dominant in postnatal skeletal muscle differentiation. It is responsible for regulating expansion, maintenance, and proliferation of SCs. It is also involved in maintaining SCs in an undifferentiated state. The combined action of Pax3 and Pax7 optimizes the conversion of stem cells into muscle cells [22,23,24].

iPSC-derived myogenic progenitors can be further differentiated to myoblasts, followed by differentiation to myocytes and the formation of multinucleated myotubes, which serve as an in vitro model for mature skeletal muscle cells. The differentiation process is closely associated with changes in transcriptional profiles. Myoblasts are usually characterized by the expression of transcription factors MyoD and Myf5, while typical markers of myotubes are myosin heavy chain (MHC), actinin, titin, or elevated desmin level.

In this study, we generated and characterized five new iPSC lines from fibroblasts obtained from EDMD1 patients and a healthy donor. These included one clone from a patient bearing an *EMD* mutation c.153del and two clones from a patient with mutation c.451dup, both causing a frameshift and creation of a premature termination codon, resulting in the lack of protein or shorter protein, respectively. Subsequently, we differentiated the newly established clones, along with four iPSC clones previously generated by reprogramming fibroblasts from two EDMD1 patients with c.153del mutation [25], into skeletal muscle cells. Using a transgene-free differentiation method, we successfully obtained cells representing three stages of skeletal muscle development: myogenic progenitors, myoblasts, and myotubes. The differentiated muscle cells expressed proper stage-specific markers. Additionally, we obtained myotubes, the final stage of in vitro muscle differentiation, showing sarcomere structure and multiple nuclei per cell, which are the features of mature skeletal muscle cells. We also observed the delayed differentiation of emerin-null cells, as those myoblasts needed more time to be able to create myotubes.

This work establishes a novel, robust model of EDMD1, providing a valuable platform for investigating disease pathogenesis and developing targeted therapies. Furthermore, the generated iPSCs may also be used in the future for the generation of cardiac and nerve cells, as well as development of multilineage organoids. The platform could be extended by preparation of isogenic controls to take into account the impact of genetic background on disease development.

## 2. Results

For iPSCs generation, we used dermal fibroblasts obtained from a skin biopsy of two EDMD1 patients and a healthy donor. Donors were male, Caucasian ethnicity, and unrelated. The patients had confirmed mutation in the *EMD* gene (*locus Xq28*) and exhibited typical EDMD symptoms [26,27,28].

We reprogrammed the fibroblasts to iPSCs using non-integrating Sendai virus (SeV) vectors encoding the four Yamanaka factors: Oct3/4, Sox2, Klf4, and c-Myc. We established two iPSC clones for the healthy donor, two clones for the patient with *EMD* mutation c.451dup, and one clone for the patient with *EMD* mutation 153del (Table 1). Additionally, we previously generated four clones from two patients with *EMD* mutation c.153del [25], which were also used in this study (Table 1).

**Figure 1:**
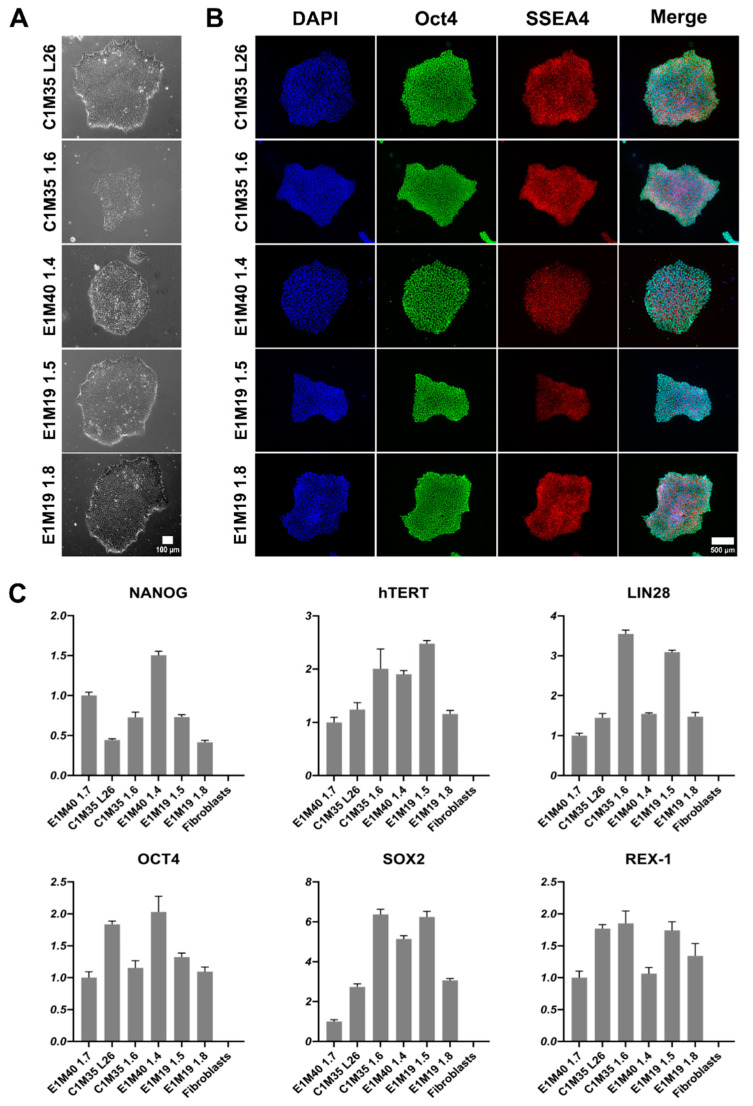
Morphology and pluripotency markers expression analysis of iPSC lines. (**A**) Images show the morphology of properly formed iPSC colonies in transmitted light (10x objective). The scale bar is 100 μm. (**B**) IF staining of iPSCs shows proper localization and equal distribution of pluripotency markers Oct4 (AF488) in nucleus and SSEA4 (TRITC) in cell surface of representative iPSC colonies. Control negative staining of HeLa cells is shown in Supplementary Figure S1. The scale bar is 500 μm. (**C**) qPCR analysis shows relative gene expression levels of iPSC pluripotency markers, normalized to the previously published E1M40 1.7 clone [25] and to glyceraldehyde-3-phosphate dehydrogenase (*GAPDH*) expression level. Error bars present standard deviations, *n* = 4 (technical replicates).

iPSCs were expanded to at least passage 10, characterized, and validated, as summarized in Table 2. Other iPSC clones were discarded due to detection of genomic instability. The morphology of colonies was typical for iPSCs [29,30,31]. The iPSC colonies had well-defined edges. They were bright and shiny under phase-contrast microscopy. Cells were round, tightly packed, and had large nuclei (Figure 1A). We confirmed the expression of pluripotency markers using immunofluorescence (IF) staining (Figure 1B; control negative staining shown in Supplementary Figure S1) and quantitative PCR (qPCR, Figure 1C). All clones expressed *OCT4*, *SSEA4*, *NANOG*, *hTERT*, *LIN28*, *SOX2*, and *REX-1*.

**Figure 2:**
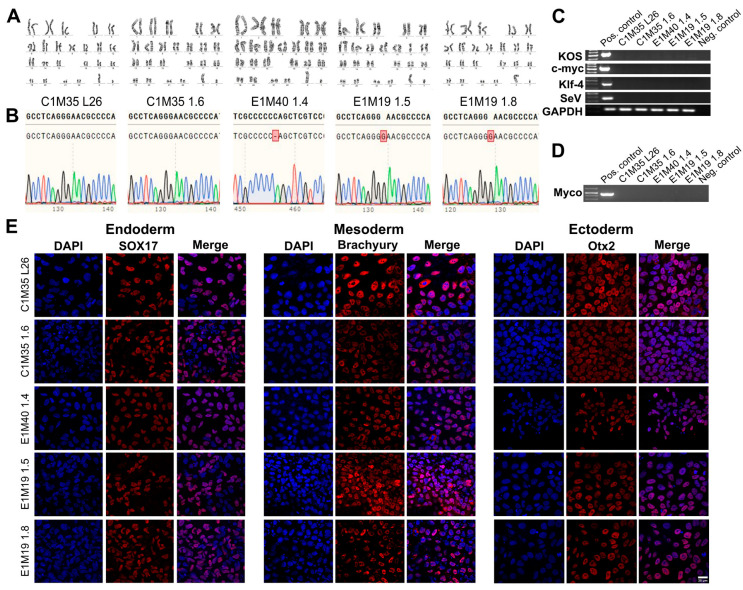
Validation of the iPSC lines—genetics, purity, and differentiation ability. (**A**) G-banding analysis shows proper 46, XY karyotypes. (**B**) DNA sequencing chromatogram of fragment of amplified *EMD* gene confirmed wild-type sequence in control lines, cytidine deletion c.153del (CCCCC_AG) in E1M40 1.4 clone, and guanine duplication c.451dup (GGG**G**AAC) in both E1M19 lines. The upper sequence shows the reference wild-type *EMD*, while the bottom sequence shows the results of DNA sequencing. Red square indicates the mutation position. (**C**) PCR verification shows the loss of each reprogramming vector (KOS, c-MYC, KLF4, SeV) in iPSC lines, together with positive control GAPDH. (**D**) PCR analysis shows lack of mycoplasma contamination in each iPSC clone. (**E**) IF staining shows germ layers markers. Each iPSC clone was differentiated independently into three germ layers on coverslips, followed by cells staining for adequate markers: Sox17 (Cy5) for endoderm, Brachyury (Cy5) for mesoderm, and Otx2 (Cy5) for ectoderm. Control negative staining of undifferentiated iPSCs is shown in Supplementary Figure S3. The scale bar is 25 μm.

The genomic stability of iPSC clones was confirmed by karyotypes analysis (Figure 2A) and short tandem repeats (STRs) profiling (16 loci, Supplementary Table S1), compared to parental fibroblasts (Supplementary Figure S2A). DNA sequencing of the *EMD* gene confirmed the wild-type sequence in control cells and the *EMD* mutations in EDMD1 cells (Figure 2B), matching the sequences found in the parental fibroblasts (Supplementary Figure S2B).

The absence of SeV vectors used for reprogramming was confirmed around passage 15 by PCR detecting four different sequences of vectors (Figure 2C). All cells were confirmed by PCR as free of mycoplasma (Figure 2D). Finally, we checked the ability of our clones to differentiate in vitro into three germ layers, using dedicated growth media (as described in Section 4). The lineage commitment was evaluated by IF staining for the following germ layers markers: Sox17 for endoderm, Brachyury for mesoderm, and Otx2 for ectoderm (Figure 2E; control negative staining of undifferentiated iPSCs is shown in Supplementary Figure S3). All tests confirmed the pluripotency, purity, and stability of the five iPSC clones.

**Figure 3:**
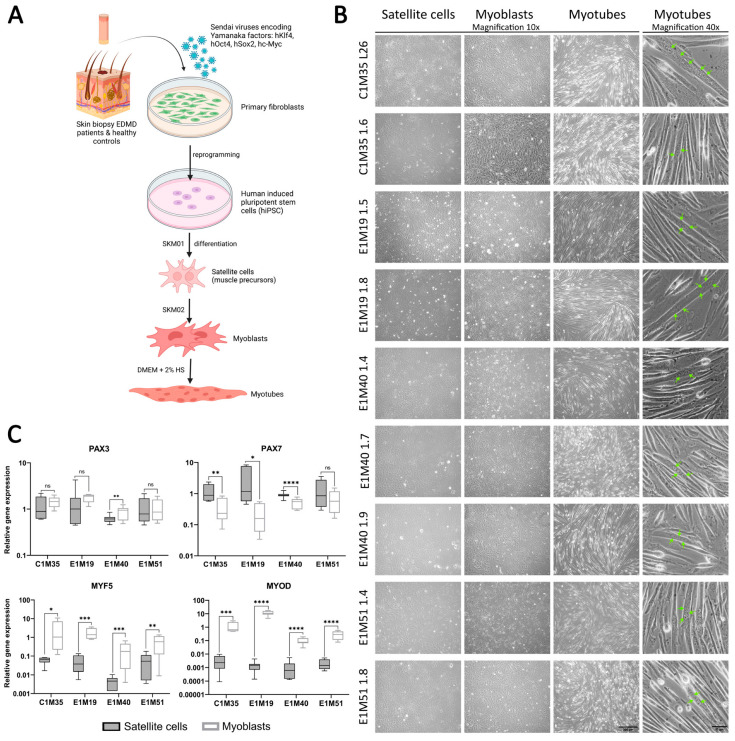
iPSCs differentiation into subsequent developmental stages of muscle cells. (**A**) Graphical representation of the reprogramming of fibroblasts from skin biopsies and differentiation process to subsequent developmental stages of muscle cells. (**B**) Images of SCs (day 6–7), myoblasts (day 6–7), and myotubes (up to 5 days in horse serum (HS)); transmission light, 10x magnification, scale bar 300 µm; with multinucleated myotubes, 40x magnification, scale bar 50 µm. Green arrows point to nuclei within an exemplary multinucleated myotube. (**C**) qPCR analyses of relative transcript levels of muscle early markers (Pax3, Pax7, Myf5, MyoD) in SCs (day 5–6) and myoblasts (day 6–7). Boxes depict 25th to 75th percentiles of relative gene expression with mean value marked with horizontal line, whiskers illustrate minimal and maximal values, asterisks indicate statistical significance (* *p* ≤ 0.05, ** *p* ≤ 0.01, *** *p* ≤ 0.001, **** *p* ≤ 0.0001, ns—not significant). Samples were normalized to GAPDH and HPRT and referred to C1M35 donor cell lines; to SCs for Pax3 and Pax7; and to myoblasts for Myf5 and MyoD, *n* ≥ 12 (≥4 biological replicates, 3 technical replicates).

As newly established clones were validated as functional iPSCs, we utilized them to obtain muscle cells in vitro. Using a transgene-free method based on growth media, we differentiated iPSCs into muscle precursors (SCs), myoblasts, and multinucleated myotubes (Figure 3A,B). In addition to the iPSCs prepared in this study, the previously published clones E1M40 1.7, E1M40 1.9, E1M51 1.4, and E1M51 1.8 [25], which have the *EMD* mutation c.153del resulting in the absence of emerin, were also differentiated to muscle cells.

**Figure 4:**
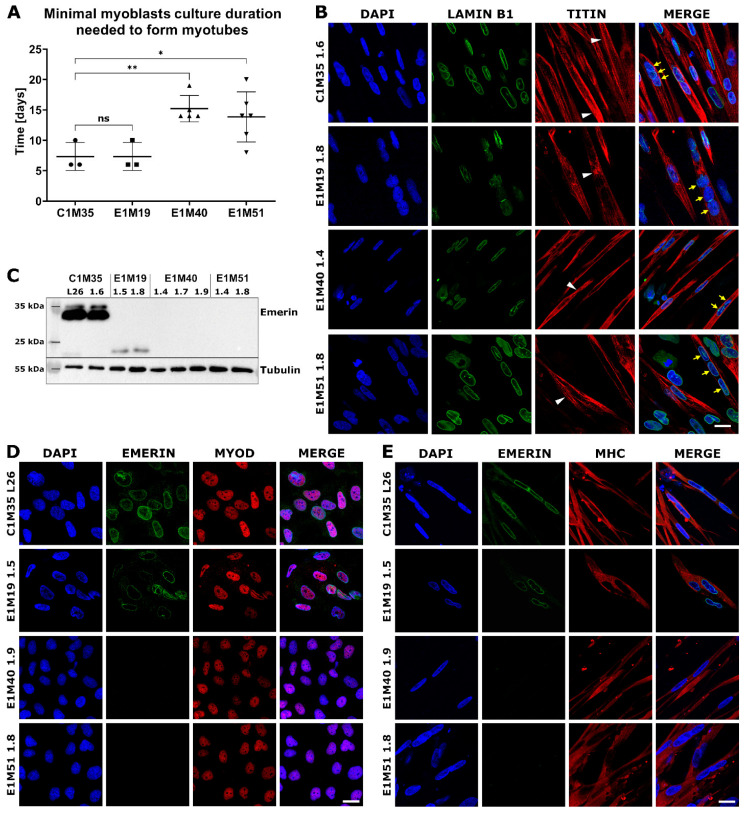
Characterization of myoblasts and myotubes obtained by iPSCs differentiation in vitro. (**A**) Graph shows the minimal duration of myoblasts culture that is necessary for ability to form myotubes in subsequent differentiation medium. Each point indicates time spent by myoblasts in myoblast medium before performing successful differentiation to myotubes. The mean is marked with a horizontal line, error bars show standard deviations, and asterisks indicate statistical significance (ns—not significant, * *p* ≤ 0.05, ** *p* ≤ 0.01); *n* ≥ 3. (**B**) IF images show myotubes derived from patients’ cells, triple-stained for chromatin (DAPI), lamin B1 (AF488), and titin (DyLight650). Titin staining shows sarcomere structure in myotubes (white arrowheads), while yellow arrows point to nuclei within an exemplary multinucleated myotube. The scale bar is 20 μm. (**C**) WB analysis of emerin level and size in myotubes. Full-length emerin was present in control cells, a lower level of its truncated form was detected in E1M19 clones, while emerin was absent in E1M40 and E1M51 myotubes. IF images of myoblasts (**D**) and myotubes (**E**) triple-stained for chromatin (DAPI), emerin (AF488), and muscle markers: MyoD (TRITC) for myoblasts and MHC (TRITC) for myotubes, confirming differentiation stage. Muscle cells show weaker signal for emerin in E1M19, and absence of signal in E1M40 and E1M51 clones. The scale bar is 20 μm.

Using qPCR, we confirmed the expression of muscle markers of particular stages of myogenesis. SCs express transcription factors Pax3 and Pax7 (Figure 3C), but not pluripotency marker Oct4 (analyzed by Western blotting (WB); see Supplementary Figure S4). At the myoblast stage, the expression levels of myoblasts markers Myf5 and MyoD were significantly elevated in comparison to SCs (see Figure 3C, at least 10-fold and 150-fold increase for Myf5 and MyoD, respectively). The Pax3 expression level was sustained in myoblasts except E1M40, while the Pax7 level decreased significantly (at least two-fold) in all donors but E1M51. Pax3, Pax7, and Myf5 presence was also confirmed by WB (Supplementary Figure S4). For each clone, we obtained elongated myotubes (Figure 3B, right panel), but the differentiation efficiency differed between samples. Depending on the clone, cells required 1–3 weeks of proliferation in myoblast medium, followed by differentiation, to be able to produce multinucleated myotubes. The prolonged culture in myoblast medium was necessary for E1M40 and E1M51 myoblasts as they did not differentiate into myotubes directly after reaching full confluency in myoblast medium, as C1M35 and E1M19 cells (Figure 4A).

Myotubes had an elongated morphology as expected, with the fraction of multinucleated cells (Figure 3B). IF analysis of myotubes revealed a sarcomere structure shown by titin bands in some cells (Figure 4B). Lamin B1 staining showed multiple nuclei in titin-positive cells, which confirms the cells’ fusion. Using WB, we confirmed the presence of typical myotube markers: desmin, actinin, and MHC. All clones expressed lamins A/C, which is the main interaction partner of emerin, and its level increases during the differentiation (Supplementary Figure S5). In conclusion, we obtained mature myotubes from control and patients-derived cells.

Using WB and IF, we evaluated the localization and level of emerin in myoblasts and myotubes. In WB analysis (Figure 4C), the full-length emerin (254 amino acids, 29 kDa, migrating around 34 kDa due to posttranslational modifications) was detected only in control cells. For E1M19 cells we noted a lower level of a faster migrating form (less than 25 kDa) of truncated emerin, resulting from a frameshift from Glycine 151, followed by a STOP codon after 208 amino acids [32]. For clones E1M40 and E1M51, we did not observe any signal, suggesting a complete loss of expression of this protein as a result of mutation c.153del. We also analyzed emerin localization in myoblasts and myotubes. In comparison to control cells C1M35, the intensity of the fluorescent signal for emerin was notably reduced in NE for E1M19 and completely disappeared in E1M40 and E1M51 cells (Figure 4D,E). All analyzed clones showed positive staining for the myoblast marker MyoD or myotubes marker MHC, respectively, confirming their muscle phenotype (Figure 4D,E).

## 3. Discussion

EDMD1, a rare genetic disorder belonging to laminopathies, still remains without a cure. Available treatments are limited to symptom-targeted therapies [33]. The precise molecular mechanisms underlying EDMD1 are not fully understood. The development of an appropriate research model is crucial for advancing our understanding of the disease, which may also lead to the discovery of more effective therapies.

iPSC-based models have been established for a range of muscular dystrophies and laminopathies, including Limb–Girdle muscular dystrophy [34], Duchenne muscular dystrophy [35], Hutchinson–Gilford progeria syndrome, or dilated cardiomyopathy [36], and also for EDMD type 2 (laminopathy caused by a mutation in the *LMNA* gene encoding lamin A/C) [37,38]. These models are widely used for in vitro differentiation into various tissues such as skeletal muscles, cardiomyocytes, adipocytes, and vascular smooth muscle cells, which serve as a model of tissues affected by a particular disease. Their utility in studying molecular mechanisms, testing candidate therapies, and exploring novel therapeutics is invaluable [34,36,38]. However, an iPSC-based model for EDMD1 has not yet been developed.

Here, we present the first cellular model of EDMD1 based on iPSCs generated from skin fibroblasts obtained from EDMD1 patients, which was verified for proper differentiation to skeletal muscle cells. This model includes a collection of iPSC lines from three patients, two of whom are unrelated but share the same mutation. This is one of the most comprehensive iPSC-based models of laminopathies, ensuring replicates of clones and control cells reprogrammed simultaneously with the patient’s cells. All clones, including those described here for the first time and previously published by our group [25], have been fully characterized and validated, demonstrating the expected functional properties of iPSCs. Additionally, the cell collection consists of phenotypes of both emerin-null and truncated emerin, enabling future studies of EDMD1 in the context of its genetic and phenotypic heterogeneity.

iPSCs may serve as a valuable starting point for various applications. In the case of EDMD1, the tissues most affected by the disease are skeletal muscles and the heart. In this work, we demonstrated the ability of iPSCs, which were generated by us, to differentiate through all stages of myogenesis, including SCs, myoblasts, and myotubes. This model allows for future in-depth analysis of myogenesis, which is difficult to achieve using other methods, particularly given the challenges associated with obtaining muscle biopsies from patients suffering from rare muscular dystrophies. The use of a transgene-free reprogramming approach minimizes potential artifacts from exogenous protein expression or genetic modifications, ensuring the disease mechanism remains unchanged.

An unexpected outcome obtained in our analysis was the sustained expression level of transcription factor Pax3 during the myoblast stage in comparison to SCs. Pax3 and Pax7 are typically considered as SCs markers, and their level usually decreases in myoblasts. However, their persistence in myoblasts has been also observed in other studies, e.g., in myoblasts and myotubes obtained by MyoD overexpression [19], or in fusing myoblasts [39]. In the model described here, the presence of Pax3 in myoblasts did not impair their differentiation ability to myotubes.

We observed that emerin-null myoblasts had delayed differentiation timing, as they needed twice-longer exposure to myoblast medium before they could successfully differentiate into myotubes (Figure 4A). This suggests that our model reflects disease phenotypes. This variability in efficiency could be investigated further to determine the molecular mechanisms and link with altered emerin expression. In the previous studies utilizing different EDMD1 models, the alterations in multiple signaling pathways important for myogenesis and muscles regeneration, such as AKT, Wnt/β-catenin, MAPK/Erk, IGF-1, TGF-β, or Notch, were associated with mutations in the *EMD* gene [5,7,40,41]. Our model allows verifying these changes in pathways’ activity using cells with patient-specific genetic background at different stages of myogenesis. Such analysis may be a starting point for further explanation of the phenomenon of delayed differentiation observed by us.

In summary, to the best of our knowledge, we have established the first iPSC-based model of EDMD1, derived from three patients, that can successfully progress through multiple stages of myogenesis. These iPSCs also have the potential for differentiation into other tissues, including neurons, cardiomyocytes, and organoid skeletal muscle models. This new EDMD1 platform provides a valuable tool for advancing therapeutic development, including high-throughput pharmacological screening, as well as gene and cell therapy investigations.

## 4. Materials and Methods

### iPSCs Generation and Expansion

Patients’ cells (fibroblasts from skin biopsies) were obtained from Dr hab. Agnieszka Madej-Pilarczyk, Mossakowski Medical Research Institute, Polish Academy of Sciences, Warsaw, Poland. Fibroblasts were cultured in a fibroblast medium containing DMEM with high glucose 4.5 g/L (Gibco, Waltham, MA USA) supplemented with 10% fetal bovine serum (FBS, Gibco), 2 mM GlutaMAX (Gibco), penicillin (100 U/mL, Gibco), and streptomycin (100 μg/mL, Gibco). For reprogramming, fibroblasts were plated on 35 mm dishes for 2 days and then transduced with a set of non-integrating Sendai viruses (SeVs) using the CytoTune 2.0 Sendai reprogramming kit (Invitrogen, Waltham, MA, USA) according to the manufacturer’s instructions. The medium was changed daily with the fresh fibroblast medium. On day 7, cells were plated on new dishes coated with Geltrex (Gibco). Starting the following day, the fibroblast medium was replaced with Essential 8 Medium (Gibco) every day. After 3–4 weeks individual iPSCs colonies were manually picked and transferred to new Geltrex-coated 24-well plates. iPSCs were expanded in Essential 8 Medium on Geltrex-coated dishes; they were dissociated with 0.5 mM EDTA in DPBS and split every 4–5 days at a ratio 1:8–1:12 using RevitaCell Supplement (Gibco). Fibroblasts and early-passage iPSCs were cultured at 37 °C in 5% CO_2_ and frozen at passage 4–5. Cells were expanded until at least passage 10 in 10% O_2_, 37 °C, 5% CO_2_, and characterized between passages 13–20.

### 4.2. In Vitro Differentiation to Three Germ Layers

In vitro trilineage differentiation was carried out using the Human Pluripotent Stem Cell Functional Identification Kit (R&D Systems, Minnneapolis, MN, USA) according to the manufacturer’s instructions. Briefly, iPSCs were trypsinized and seeded on Geltrex-coated coverslips in a 24-well plate, at a density of approximately 9 × 10^4^ cells per well in Essential 8 Medium with RevitaCell Supplement.

After obtaining 60–70% confluence for mesoderm and 70–80% for ectoderm and endoderm (24 h/48 h), E8 Medium was replaced with proper differentiation medium (for endoderm, ectoderm, or mesoderm, R&D Systems), and after 40 h for mesoderm and 72 h for ectoderm and endoderm coverslips were fixed with 4% PFA and stained for differentiation markers (as described in Section 4.4).

### 4.3. Differentiation of iPSCs into Skeletal Muscle Cells

To generate skeletal muscle cells, the iPSC lines were seeded in 60 mm dishes coated with Geltrex, at a density of 2500 or 5000 cells per 1 cm^2^, depending on the clone, in Skeletal Muscle Induction Medium (SKM01, AMSBIO) supplemented with penicillin-streptomycin (Gibco). For the first 24 h, the RevitaCell Supplement was added to the medium. Then, the medium was changed every other day. Upon reaching full confluency (5–7 days), cells were collected using TrypLE Express Enzyme (Gibco) as SCs and seeded at a density of 2500 cells per 1 cm^2^ of a 60 mm dish coated with Geltrex. Cells were seeded in the myoblast medium (SKM02, AMSBIO, Abingdon, UK) supplemented with penicillin-streptomycin to induce differentiation into myoblasts. The medium was changed every 2–3 days. After the next 6–8 days, at the point of full confluency, the medium was changed to SKM03+ (AMSBIO) or DMEM-HS (DMEM high glucose (4.5 g/L, Gibco) supplemented with 2% HS (Gibco)), GlutaMAX Supplement, and penicillin-streptomycin for myotube formation, and the cells were collected as myotubes after 3–7 days. Some clones needed additional culture time in SKM02 (up to 3 weeks), with passages every 3–5 days upon reaching full confluency, to be able to differentiate into myotubes. Cells were cultured in standard conditions (37 °C, 5% CO_2_). Upon each differentiation stage cells were imaged with ZEN Software version 3.8 on a ZEISS AxioVert microscope (Zeiss) with 10x objective and for myotubes additionally with 40x objective.

### 4.4. Short Tandem Repeats (STRs) Analysis

The profiling of the human cell lines was performed by Microsynth and Eurofins companies (Balgach, Switzerland) using highly polymorphic STR loci. They were amplified using the PowerPlex 16 HS System (Promega, Madison, WI, USA). Fragment analysis was performed on an ABI3730xl (Life Technologies, Waltham, MA, USA) and the resulting data were analyzed with GeneMarker HID software (Softgenetics, State College, PA, USA).

Eurofins performs genotyping according to ANSI/ATCC standard ASN-0002 testing 16 DNA markers using the Applied Biosystems AmpFLSTR Identifiler Plus PCR Amplification Kit system.

### 4.5. Immunofluorescence (IF)

iPSCs were seeded on Geltrex-coated glass coverslips and cultured for 3–4 days until proper-sized colonies were formed. Myoblasts on Geltrex-coated glass coverslips in SKM02 medium were cultured 4–8 days to about 80% confluency when coverslips were fixed. To obtain myotubes specimens, myoblasts were seeded on Geltrex-coated glass coverslips and cultured for 3–4 days until reaching 100% confluency. Then, the medium was changed to DMEM-HS or SKM03 and after 3–7 days myotubes were formed. Then, coverslips were fixed in 4% paraformaldehyde for 20 min, washed with PBS, permeabilized with 0.5% Triton X-100 for 5 min at room temperature (RT), and washed again with PBS. Preparations were blocked for 30 min at RT with 1% donkey serum (DS, Gibco) or 1% FBS (Gibco) in PBS for germ layer markers and other stainings, respectively. Then, preparations were incubated with primary unconjugated antibodies overnight at 4 °C, washed with PBS, and incubated with secondary antibodies or primary antibodies conjugated with a fluorophore for 1 h at RT, and washed again with PBS. Antibodies for iPSCs and germ layer markers were diluted in 1% DS in PBS (antibodies and dilutions used shown in Table 3). Coverslips were mounted on glass slides with the DABCO mounting medium (Fluka, Paris, France) with DAPI. Pluripotency markers were visualized with a ZEISS AxioVert A1 fluorescence microscope using a 10x objective, using ZEN Software. Myoblasts, myotubes, and germ layers staining was visualized with an SP8 (Leica,) or Stellaris (Leica) microscope using a 63x oil objective.

### 4.6. Gene Expression Analysis

Total RNA was extracted from iPSCs using the Universal DNA/RNA/Protein Purification Kit (EurX, Warsaw, Poland). An amount of 1 μg of total RNA was reverse-transcribed to cDNA with the Maxima First Strand cDNA Synthesis Kit for RT-qPCR (Thermo Scientific, Waltham, MA, USA). Real-time qPCR was performed using SYBR Green Master Mix (Applied Biosystems, Foster City, CA, USA) and Quant Studio 5 Real-Time PCR System (Applied Biosystems); all samples were analyzed in four technical replicates. The reaction parameters were as follows: 50 °C for 2 min, 95 °C for 2 min, followed by 40 cycles of denaturation at 95 °C for 15 s and annealing/extension at 64 °C for 30 s. Additionally, the 3-step melt curve was performed (95 °C for 15 s, 60 °C for 1 min, 95 °C for 15 s). Pluripotency markers’ relative gene expression was normalized to Glyceraldehyde-3-Phosphate Dehydrogenase (*GAPDH*) and referred to an established iPSCs line E1M40 1.7 (UWRBTi003-B [25]). The sequences of the designed primers are listed in Table 4.

For the analysis of muscle markers, the total RNA was extracted using the Direct-zol RNA Microprep Kit (Zymo Research, Irvine, CA, USA). An amount of 500 ng of the isolated RNA was reverse-transcribed as described above. qPCR was performed as described with samples being analyzed in at least 4 biological replicates, each in three technical repeats. Relative gene expression was normalized to GAPDH and hypoxanthine phosphoribosyltransferase (HPRT) and referred to C1M35 donor cell lines (to C1M35 SCs for Pax3 and Pax7 and to C1M35 myoblasts for Myf5 and MyoD). Primers’ sequences are presented in Table 4. Statistical significance was calculated using multiple *t*-tests in GraphPad Prism (version 8.4.3).

### 4.7. Sequencing

Genomic DNA was isolated using the Universal DNA/RNA/Protein Purification Kit (EurX), then the *EMD* gene was amplified with Phusion HSII DNA Polymerase (Thermo Scientific), with GC buffer, 3% DMSO, and previously published [16] primers listed in Table 4. Reactions were performed using thermocycler TOne 96G (Biometra, Göttingen, Germany) with the following parameters: 98 °C for 5 min, followed by 35 cycles of denaturing at 98 °C for 15 s, annealing at 62 °C for 30 s, extension at 72 °C for 90 s, and 10 min final extension at 72 °C. PCR product was purified with the PCR/DNA Clean-Up Purification Kit (EurX) and sequenced by Microsynth company with sequencing primers shown in Table 4.

### 4.8. Karyotyping

iPSC lines and fibroblasts were treated with 0.67 μg/mL colcemid (BioWest, Nuaillé, France) for 2.5 h, then dissociated by Trypsin-EDTA (Gibco) and centrifuged at 1400 rpm, 7 min. After incubation in hypotonic solution (Ohnuki’s solution) and fixation in methanol:glacial acetic acid (3:1) mixture (Chempur, Karlsruhe, Germany), microscopic slides were prepared. G-banded metaphase analysis was performed according to the International System for Human Cytogenomic Nomenclature 2020 (ISCN 2020; AGT manual [42,43]) employing the Imager.M1 (Zeiss) microscope and the Ikaros software (version 6.3, Metasystems DE, Altlussheim, Germany). Up to 30 metaphases from iPSCs (resolution 400–450 bands) and at least 15 metaphases from fibroblasts (resolution 350–400) were karyotyped by an experienced cytogeneticist.

### 4.9. The Episomal Vectors’ Presence

Episomal vectors’ presence was analyzed by PCR performed for iPSCs cDNA using 5 pairs of primers shown in Table 4, four pairs recognizing reprogramming vectors (sequences from CytoTune-iPS 2.0 Sendai Reprogramming Kit), and 1 pair for *GAPDH* as control. Reactions were performed with Taq polymerase (EURx), with 35 cycles of amplification, using thermocycler TOne 96G (Biometra) with the following parameters: 95 °C for 5 min, followed by 35 cycles of denaturing at 95 °C for 30 s, annealing at 55 °C (*c-MYC*, *GAPDH*, *SeV*, *KOS*) or 63 °C (*KLF4*) for 30 s, extension at 72 °C for 30 s, and 5 min final extension at 72 °C. Normal human dermal fibroblasts (Lonza, Basel, Switzerland) were used as a negative control, and cells collected shortly after SeV transduction were used as a positive control.

### 4.10. Mycoplasma Test

A mycoplasma PCR test was performed on culture supernatants (at least 48 h without medium change and confluence at least 80%) using the Mycoplasma PCR Detection Kit (Abcam, Cambridge, UK) according to the manufacturer’s instructions.

### 4.11. Western Blotting (WB)

Cell pellets were resuspended in hot Laemmli lysis buffer (50 mM Tris-HCl pH 6.8; 10% glycerol; 2% SDS). Lysates were boiled for 5 min (96 °C) and cooled. BCA assay (Thermo Scientific) was performed for 10x diluted samples, according to the manufacturer’s instructions, in a 96-well-plate format. After measurements, samples were supplemented with DTT to a final concentration of 50 mM and boiled again. An amount of 25 µg of protein extracts were separated via SDS-PAGE on 6–12% gels, transferred to nitrocellulose membranes (0.45 μm pore sizes), and blocked in 5% non-fat milk in PBS with 0.075% Tween 20 (PBST) for 1 h, RT. Primary antibodies were applied overnight at 4 °C in PBST (or in 5% non-fat milk as marked in the Table 3), and secondary antibodies conjugated with HRP were applied for 1 h, RT in 5% milk in PBST buffer (antibodies and dilutions shown in Table 3). The proteins were visualized using ECL substrate (Bio-Rad, Hercules, CA, USA) and analyzed with ImageLab 5.0 (Bio-Rad).

## Figures and Tables

**Table 1:** Summary of generated iPSC clones. The table summarizes newly validated iPSC clones from three donors alongside previously reported clones from two donors. Emerin protein level was evaluated by WB analysis in myotubes (Figure 4C). Bold letter in EMD sequence - mutations’ site.

iPSCs Line Name	Unique Stem Cell Line Identifier	Disease	Age of Biopsy	Genotype (*EMD* Gene)	Emerin Expression
**Newly established clones**					
C1M35 L26	UWRBTi001-C	-	35	NM_000117.3(EMD), CCCCC**C**AGC, GG**G**AACGCC	normal
C1M35 1.6	UWRBTi001-D	-			
E1M40 1.4	UWRBTi003-A	EDMD1	40	NM_000117.3(EMD):c.153del, p.(Ser52fs), CCCCC_AGCT, hemizygous, rs876661345	not detected
E1M19 1.5	UWRBTi005-A	EDMD1	19	NM_000117.3(EMD):c.451dup *, p.(Glu151fs), GGG**G**AACGCC, hemizygous	truncated protein, lower level
E1M19 1.8	UWRBTi005-B	EDMD1			
**Previously generated clones**					
E1M40 1.7	UWRBTi003-B	EDMD1	40	NM_000117.3(EMD):c.153del, p.(Ser52fs), CCCCC_AGCT, hemizygous, rs876661345	not detected
E1M40 1.9	UWRBTi003-C	EDMD1			
E1M51 1.4	UWRBTi004-A	EDMD1	51	NM_000117.3(EMD):c.153del, p.(Ser52fs), CCCCC_AGCT, hemizygous, rs876661345	not detected
E1M51 1.8	UWRBTi004-B	EDMD1			

* Mutation c.451dup was previously reported also as c.450insG or c.450dup (e.g., www.umd.be/emd database (accessed on 4 February 2025)).

**Table 2:** Characterization and validation of iPSCs. The table contains all tests performed for iPSCs validation with reference to corresponding data.

Parameter	Test Results	Data
**Colony morphology**	Confirmation of normal colony morphology with transmission light microscopy	Figure 1A
**Phenotype**	IF confirmation of pluripotency markers presence (Oct4, SSEA4)	Figure 1B
	qPCR detection of pluripotency markers expression (*NANOG*, *hTERT*, *LIN28*, *OCT4*, *SOX2*, *REX-1*)	Figure 1C
**Genotype**	46, XY karyotype confirmed by G-band analysis	Figure 2A, Supplementary Figure S2A
**Identity**	STRs analysis (16 loci, all matched)	Supplementary Table S1
**Mutation analysis**	DNA sequencing of amplified *EMD* confirmed wild-type gene or mutation (according to Table 1)	Figure 2B, Supplementary Figure S2B
**Reprogramming factors presence**	The PCR test for reprogramming factors was negative	Figure 2C
**Microbiology**	PCR test for mycoplasma was negative	Figure 2D
**Differentiation potential**	Directed differentiation followed by positive staining for three germ layers: Sox17 (endoderm), Brachyury (mesoderm), Otx2 (ectoderm)	Figure 2E

**Table 3:** Antibodies used for immunofluorescence (IF) and Western blotting (WB) staining.

	Antibody	Dilution	Company, Cat#
Pluripotency markers	Rabbit anti Oct4 Mouse anti SSEA4 DyLight550	1:100 IF/1:1000 WB 1:100 IF	Cell Signaling Technology cat# 2750 Invitrogen cat# MA1-021-D550
iPSCs differentiation markers	Goat anti SOX17 Goat anti Otx2 Goat anti Brachyury	1:10 IF 1:10 IF 1:10 IF	R&D Systems cat# AF1924 R&D Systems cat# AF1979 R&D Systems cat# AF2085
Skeletal muscle markers and reference proteins	Mouse anti Pax3 Mouse anti Pax7 Mouse anti MyoD Rabbit anti Myf5 Mouse anti MHC Mouse anti desmin Mouse anti actinin Mouse anti myogenin Rabbit anti GAPDH Mouse anti actin α Mouse anti tubulin α Rabbit anti emerin Rabbit anti lamin A/C Rabbit anti lamin B1	1:1000 WB 1:200 WB (milk) 1:30 IF 1:2500 WB (milk) 1:10 IF/1:500 WB 1:2500 WB 1:2000 WB (milk) 1:50 WB 1:10000 WB (milk) 1:1000 WB 1:1000 WB (milk) 1:400 IF/1:1000 WB (milk) 1:4000 WB (milk) 1:100 IF/1:500 WB	DSHB clone C2 [D1] DSHB [D2] Santa Cruz cat# sc-377460 Abcam cat# ab125078 DSHB clone MF-20 [D3] Sigma Aldrich cat# SAB4200707 Sigma Aldrich cat# A7811 DSHB clone F5D [D4] Sigma Aldrich cat# G9545 Sigma Aldrich cat# A2228 DSHB clone 12G10 [D5] Cell Signaling Technology cat# 30853 Cell Signaling Technology cat# 2032S Proteintech cat# 12987-1-AP
Secondary antibodies	Donkey anti-goat AF568 Donkey anti-rabbit TRITC Goat anti-mouse IgM DyLight650 Donkey anti-rabbit AF488 Goat anti-rabbit HRP Donkey anti mouse HRP	1:200 IF 1:50 IF 1:50 IF 1:200 IF 1:10,000 WB 1:10,000 WB	Invitrogen cat# A11057 Jackson Immunoresearch cat# 711-025-152 Invitrogen cat# SA5-10153 Jackson Immunoresearch cat# 711-545-152 Jackson Immunoresearch cat#111-035-144 Jackson Immunoresearch cat# 715-035-151

Antibodies deposited to the Developmental Studies Hybridoma Bank (DSHB) by [D1] C.P. Ordahl, [D2] A. Kawakami, [D3] D.A. Fischman, [D4] W.E. Wright, and J. Frankel/E.M. Nelsen. MHC—myosin heavy chain, HRP—horseradish peroxidase.

**Table 4:** Primers used for qPCR, *EMD* sequencing, and episomal vector presence detection. SeV sequence is a genomic sequence of Sendai virus. *KOS* sequence contains human *KLF4, OCT3/4*, and *SOX2*.

Primers		
	Target	Forward/Reverse Primer (5′->3′)
Episomal plasmids (PCR)	SeV *KOS* *KLF4* *c-MYC*	GGATCACTAGGTGATATCGAGC/ACCAGACAAGAGTTTAAGAGATATGTATC ATGCACCGCTACGACGTGAGCGC/ACCTTGACAATCCTGATGTGG TTCCTGCATGCCAGAGGAGCCC/AATGTATCGAAGGTGCTCAA TAACTGACTAGCAGGCTTGTCG/TCCACATACAGTCCTGGATGATGATG
Pluripotency markers (qPCR)	*NANOG* *OCT4* *SOX2* *LIN28* *hTERT* *REX-1*	CCTCCAGCAGATGCAAGAACTC/CCTTCTGCGTCACACCATTGCTA AGTTTGTGCCAGGGTTTTTG/ACTTCACCTTCCCTCCAACC TGCACATGAAGGAGCACCCG/GCTCGCCATGCTATTGCCGC GAGGCAGTGGAGTTCACCTTTAAG/GATCTGCGCTTCTGCATGCTC GGAGCAAGTTGCAAAGCATTG/TCCCACGACGTAGTCCATGTT GCTGAAACAAATGTACTGAGGCTG/GCTGTCTTCAGCAAACACCTGC
Skeletal muscle markers (qPCR)	*PAX3* *PAX7* *MYF5* *MYOD*	CAAGCCCAAGCAGGTGACAAC/CACAGACCGCGTCCTTGAGTAA TTTGCCGACTTTGGATTCGTCCC/GGTGGACACTTCCAAAGGGAATC ATCGAGAGCCTGCAGGAGTTG/TACATTCGGGCATGCCATCAGAG GACGGCATGATGGACTACAGC/CGCCTCGTTGTAGTAGGCGC
House-keeping genes (qPCR)	*GAPDH* (for qPCR) *GAPDH* (for PCR) *HPRT* (for qPCR)	CGGAGTCAACGGATTTGGTC/CATGTAAACCATGTAGTTGAGGTC CGGAGTCAACGGATTTGGTC/CATGTAAACCATGTAGTTGAGGTC CCCTGGCGTCGTGATTAGTG/GCAAGACGTTCAGTCCTGTCC
Targeted mutation sequencing	*EMD* amplification *EMD* sequencing	CTCCCGCGGTTAGGTCCCG/TTCCCCAAAGACCTAGCTCTG CTCCCGCGGTTAGGTCC (for mutation c.153del) CATGACAGGGCCATGGTGG (for mutation c.451dup)

## Data Availability

The original contributions presented in this study are included in the article/Supplementary Materials and are openly available in zenodo.org at DOI 10.5281/zenodo.14810979. Further inquiries can be directed to the corresponding author(s).

## Supplementary Materials

The supporting information can be downloaded at https://www.mdpi.com/article/10.3390/ijms26041539/s1.

## References

[1] Viggiano E., Madej-Pilarczyk A., Carboni N., Picillo E., Ergoli M., del Gaudio S., Marchel M., Nigro G., Palladino A., Politano L. (2019). X-Linked Emery–Dreifuss Muscular Dystrophy: Study Of X-Chromosome Inactivation and Its Relation with Clinical Phenotypes in Female Carriers. Genes.

[2] Emery A.E. (1989). Emery-Dreifuss syndrome. J. Med. Genet..

[3] Lammerding J. (2011). Mechanics of the Nucleus. Comprehensive Physiology.

[4] Berk J.M., Tifft E.K., Wilson K.L. (2013). The nuclear envelope LEM-domain protein emerin. Nucleus.

[5] Koch A.J., Holaska J.M. (2014). Emerin in health and disease. Semin. Cell Dev. Biol..

[6] Holaska J.M. (2008). Emerin and the Nuclear Lamina in Muscle and Cardiac Disease. Circ. Res..

[7] Storey E.C., Holt I., Morris E.G., Fuller H.R. (2020). Muscle cell differentiation and development pathway defects in Emery-Dreifuss muscular dystrophy. Neuromuscul. Disord..

[8] Iyer A., Holaska J.M. (2020). EDMD-Causing Emerin Mutant Myogenic Progenitors Exhibit Impaired Differentiation Using Similar Mechanisms. Cells.

[9] Frock R.L., Kudlow B.A., Evans A.M., Jameson S.A., Hauschka S.D., Kennedy B.K. (2006). Lamin A/C and emerin are critical for skeletal muscle satellite cell differentiation. Genes Dev..

[10] Gnocchi V.F., Ellis J.A., Zammit P.S. (2008). Does satellite cell dysfunction contribute to disease progression in Emery–Dreifuss muscular dystrophy?. Biochem. Soc. Trans..

[11] Fidziańska A., Toniolo D., Hausmanowa-Petrusewicz I. (1998). Ultrastructural abnormality of sarcolemmal nuclei in Emery-Dreifuss muscular dystrophy (EDMD). J. Neurol. Sci..

[12] Park Y.-E., Hayashi Y.K., Goto K., Komaki H., Hayashi Y., Inuzuka T., Noguchi S., Nonaka I., Nishino I. (2009). Nuclear changes in skeletal muscle extend to satellite cells in autosomal dominant Emery-Dreifuss muscular dystrophy/limb-girdle muscular dystrophy 1B. Neuromuscul. Disord..

[13] Mittelbronn M., Hanisch F., Gleichmann M., Stötter M., Korinthenberg R., Wehnert M., Bonne G., Rudnik-Schöneborn S., Bornemann A. (2006). Myofiber degeneration in autosomal dominant Emery-Dreifuss muscular dystrophy (AD-EDMD) (LGMD1B). Brain Pathol..

[14] Melcon G., Kozlov S., Cutler D.A., Sullivan T., Hernandez L., Zhao P., Mitchell S., Nader G., Bakay M., Rottman J.N. (2006). Loss of emerin at the nuclear envelope disrupts the Rb1/E2F and MyoD pathways during muscle regeneration. Hum. Mol. Genet..

[15] Ozawa R., Hayashi Y.K., Ogawa M., Kurokawa R., Matsumoto H., Noguchi S., Nonaka I., Nishino I. (2006). Emerin-Lacking Mice Show Minimal Motor and Cardiac Dysfunctions with Nuclear-Associated Vacuoles. Am. J. Pathol..

[16] Brown A.C., Scharner J., Felice K., Meriggioli M.N., Tarnopolsky M., Bower M., Zammit P.S., Mendell J.R., Ellis A.J. (2011). Novel and recurrent EMD mutations in patients with Emery–Dreifuss muscular dystrophy, identify exon 2 as a mutation hot spot. J. Hum. Genet..

[17] Zhang M., Chen J., Si D., Zheng Y., Jiao H., Feng Z., Hu Z., Duan R. (2014). Whole exome sequencing identifies a novel EMDmutation in a Chinese family with dilated cardiomyopathy. BMC Med. Genet..

[18] Kong D., Zhan Y., Liu C., Hu Y., Zhou Y., Luo J., Gu L., Zhou X., Zhang Z. (2019). A Novel Mutation of The EMD Gene In A Family With Cardiac Conduction Abnormalities And A High Incidence Of Sudden Cardiac Death. Pharmgenomics Pers. Med..

[19] Benarroch L., Madsen-Østerbye J., Abdelhalim M., Mamchaoui K., Ohana J., Bigot A., Mouly V., Bonne G., Bertrand A.T., Collas P. (2023). Cellular and Genomic Features of Muscle Differentiation from Isogenic Fibroblasts and Myoblasts. Cells.

[20] Caron L., Kher D., Lee K.L., McKernan R., Dumevska B., Hidalgo A., Li J., Yang H., Main H., Ferri G. (2016). A Human Pluripotent Stem Cell Model of Facioscapulohumeral Muscular Dystrophy-Affected Skeletal Muscles. Stem. Cells Transl. Med..

[21] Xi H., Langerman J., Sabri S., Chien P., Young C.S., Younesi S., Hicks M., Gonzalez K., Fujiwara W., Marzi J. (2020). A Human Skeletal Muscle Atlas Identifies the Trajectories of Stem and Progenitor Cells across Development and from Human Pluripotent Stem Cells. Cell Stem. Cell..

[22] Hernández-Hernández J.M., García-González E.G., Brun C.E., Rudnicki M.A. (2017). The myogenic regulatory factors, determinants of muscle development, cell identity and regeneration. Semin. Cell Dev. Biol..

[23] Asfour H.A., Allouh M.Z., Said R.S. (2018). Myogenic regulatory factors: The orchestrators of myogenesis after 30 years of discovery. Exp. Biol. Med..

[24] Buckingham M., Relaix F. (2015). PAX3 and PAX7 as upstream regulators of myogenesis. Semin. Cell Dev. Biol..

[25] Machowska M., Bearzi C., Piekarowicz K., Łaczmańska I., Rzepecki R. (2021). Generation of one control and four iPSCs clones from patients with Emery-Dreifuss muscular dystrophy type 1. Stem. Cell Res..

[26] Hausmanowa-Petrusewicz I., Madej-Pilarczyk A., Marchel M., Opolski G. (2009). Emery-Dreifuss dystrophy: A 4-year follow-up on a laminopathy of special interest. Neurol. Neurochir. Pol..

[27] Niebroj-Dobosz I., Madej-Pilarczyk A., Marchel M., Sokołowska B., Hausmanowa-Petrusewicz I. (2009). Matrix metalloproteinases in serum of Emery-Dreifuss muscular dystrophy patients. Acta Biochim. Pol..

[28] Niebroj-Dobosz I., Madej-Pilarczyk A., Marchel M., Sokołowska B., Hausmanowa-Petrusewicz I. (2011). Circulating tenascin-C levels in patients with dilated cardiomyopathy in the course of Emery-Dreifuss muscular dystrophy. Clin. Chim. Acta.

[29] Cheng Y., Xu M., Chen G., Beers J., Chen C.Z., Liu C., Zou J., Zheng W. (2023). A Protocol for Culture and Characterization of Human Induced Pluripotent Stem Cells After Induction. Curr. Protoc..

[30] Swamydas M., Narayanan P., Luangphakdy V., Furyes A., Duginski G., Nakamura K., Muschler G. (2024). Characterization of IPSC colony morphology and variation to assist in automated clone picking for clonal expansion. Cytotherapy.

[31] Amit M., Itskovitz-Eldor J., Laevsky I., Novak A. (2012). Atlas of Human Pluripotent Stem Cells: Derivation and Culturing.

[32] Dubińska-Magiera M., Kozioł K., Machowska M., Piekarowicz K., Filipczak D., Rzepecki R. (2019). Emerin Is Required for Proper Nucleus Reassembly after Mitosis: Implications for New Pathogenetic Mechanisms for Laminopathies Detected in EDMD1 Patients. Cells.

[33] Heller S.A., Shih R., Kalra R., Kang P.B. (2020). Emery-Dreifuss muscular dystrophy. Muscle Nerve.

[34] Bruge C., Geoffroy M., Benabides M., Pellier E., Gicquel E., Dhiab J., Hoch L., Richard I., Nissan X. (2022). Skeletal Muscle Cells Derived from Induced Pluripotent Stem Cells: A Platform for Limb Girdle Muscular Dystrophies. Biomedicines.

[35] Danišovič L., Culenova M., Csobonyeiova M. (2018). Induced Pluripotent Stem Cells for Duchenne Muscular Dystrophy Modeling and Therapy. Cells.

[36] Crasto S., Di Pasquale E. (2018). Induced Pluripotent Stem Cells to Study Mechanisms of Laminopathies: Focus on Epigenetics. Front. Cell Dev. Biol..

[37] Perepelina K., Kostina A., Klauzen P., Khudiakov A., Rabino M., Crasto S., Zlotina A., Fomicheva Y., Sergushichev A., Oganesian M. (2020). Generation of two iPSC lines (FAMRCi007-A and FAMRCi007-B) from patient with Emery–Dreifuss muscular dystrophy and heart rhythm abnormalities carrying genetic variant LMNA p.Arg249Gln. Stem. Cell Res..

[38] Cho S., Lee C., Lai C., Zhuge Y., Haddad F., Fowler M., Sallam K., Wu J.C. (2022). Heterozygous LMNA mutation-carrying iPSC lines from three cardiac laminopathy patients. Stem. Cell Res..

[39] Brzóska E., Przewoźniak M., Grabowska I., Jańczyk-Ilach K., Moraczewski J. (2009). Pax3 and Pax7 expression during myoblast differentiation in vitro and fast and slow muscle regeneration in vivo. Cell Biol. Int..

[40] Koch A.J., Holaska J.M. (2012). Loss of Emerin Alters Myogenic Signaling and miRNA Expression in Mouse Myogenic Progenitors. PLoS ONE.

[41] Dubinska-Magiera M., Zaremba-Czogalla M., Rzepecki R. (2013). Muscle development, regeneration and laminopathies: How lamins or lamina-associated proteins can contribute to muscle development, regeneration and disease. Cell Mol. Life Sci..

[42] Lawce H.J. (2017). Chromosome stains. The AGT Cytogenetics Laboratory Manual.

[43] McGowan-Jordan J., Hastings R.J., Moore S. (2020). ISCN 2020.

