# Supplementary material for: Human iPSC-Derived Muscle Cells as a New Model for Investigation of EDMD1 Pathogenesis
Source: Int J Mol Sci. 2025 Feb 12;26(4):1539. doi: 10.3390/ijms26041539 (PMC11855679; doi:10.3390/ijms26041539)
Supplement: Supplementary file 1 [file ijms-26-01539-s001.zip › ijms-3385491-supplementary.pdf]

SUPPLEMENTARY DATA

LOCI	E1M40 FIBRO	E1M40 1.4	C1M35 FIBRO	C1M35 1.6	C1M35 L26	E1M19 FIBRO	E1M19 1.5	E1M19 1.8
D8S1179	13 15	13 15	12	12	12	12 13	12 13	12 13
D21S11	28 31	28 31	30 32.2	30 32.2	30 32.2	29 31	29 31	29 31
D7S820	9	9	11 12	11 12	11 12	11 12	11 12	11 12
CSF1PO	11 12	11 12	11 13	11 13	11 13	10 11	10 11	10 11
D3S1358	18 20	18 20	16 19	16 19	16 19	14 15	14 15	14 15
TH01	7 9	7 9	7 8	7 8	7 8	6 9	6 9	6 9
D13S317	12 13	12 13	8 12	8 12	8 12	11 12	11 12	11 12
D16S539	12	12	11	11	11	9 11	9 11	9 11
vWA	14 15	14 15	16 18	16 18	16 18	16 16	16 16	16 16
TPOX	10 11	10 11	8	8	8	8 11	8 11	8 11
D18S51	11 15	11 15	12 16	12 16	12 16	13 14	13 14	13 14
AMEL	X Y	X Y	X Y	X Y	X Y	X Y	X Y	X Y
D5S818	12	12	11 14	11 14	11 14	11 13	11 13	11 13
FGA	21 23	21 23	22 23	22 23	22 23	20 23	20 23	20 23

Table S1. STR profiles summary of characterized iPSC clones and their parental fibroblasts.

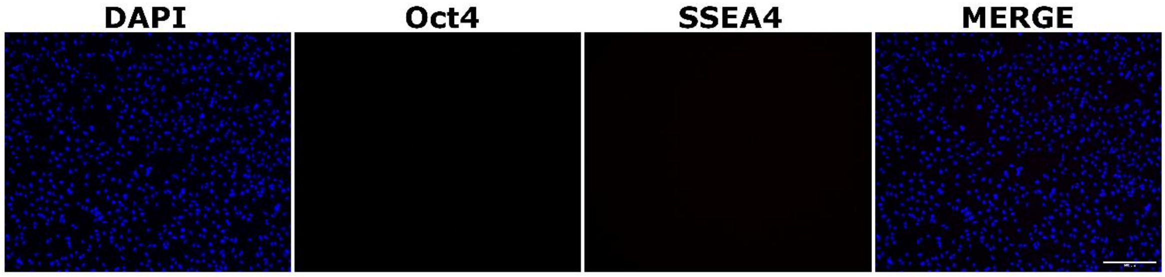

Figure S1. Negative control of staining performed for pluripotency markers (Oct4 and SSEA4) on HeLa cells. This result confirms specificity of staining in Figure 1B. Scale bar is 500 µm.

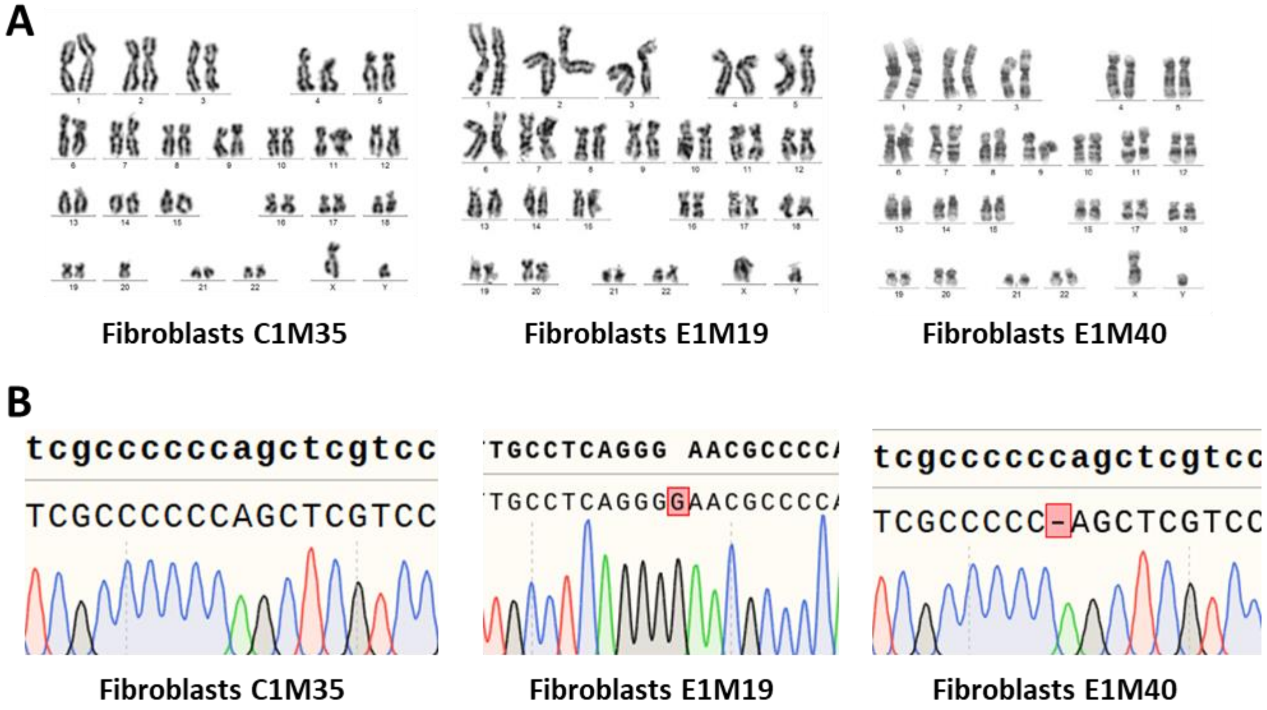

Figure S2. Analysis of parental fibroblast lines. (A) Karyotypes of parental fibroblasts. (B) DNA sequencing results of *EMD* gene in parental fibroblasts. The upper sequence shows the reference wild-type *EMD*, while the bottom sequence shows the result of DNA sequencing.

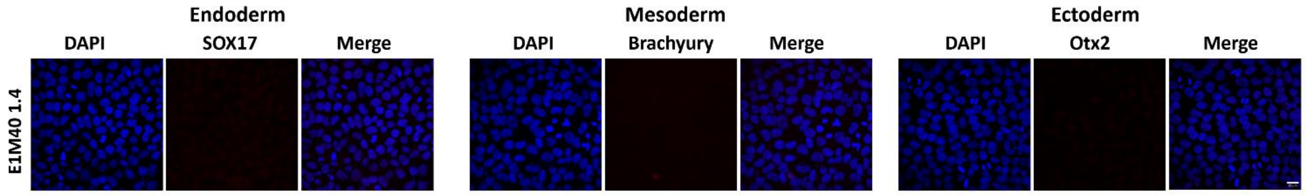

Figure S3. Negative control of staining of three germ layers markers (Sox17, Brachyury and Otx2) performed for undifferentiated iPSC clone E1M40 1.4. This result confirms specificity of staining in Figure 2E. Scale bar is 20 μm.

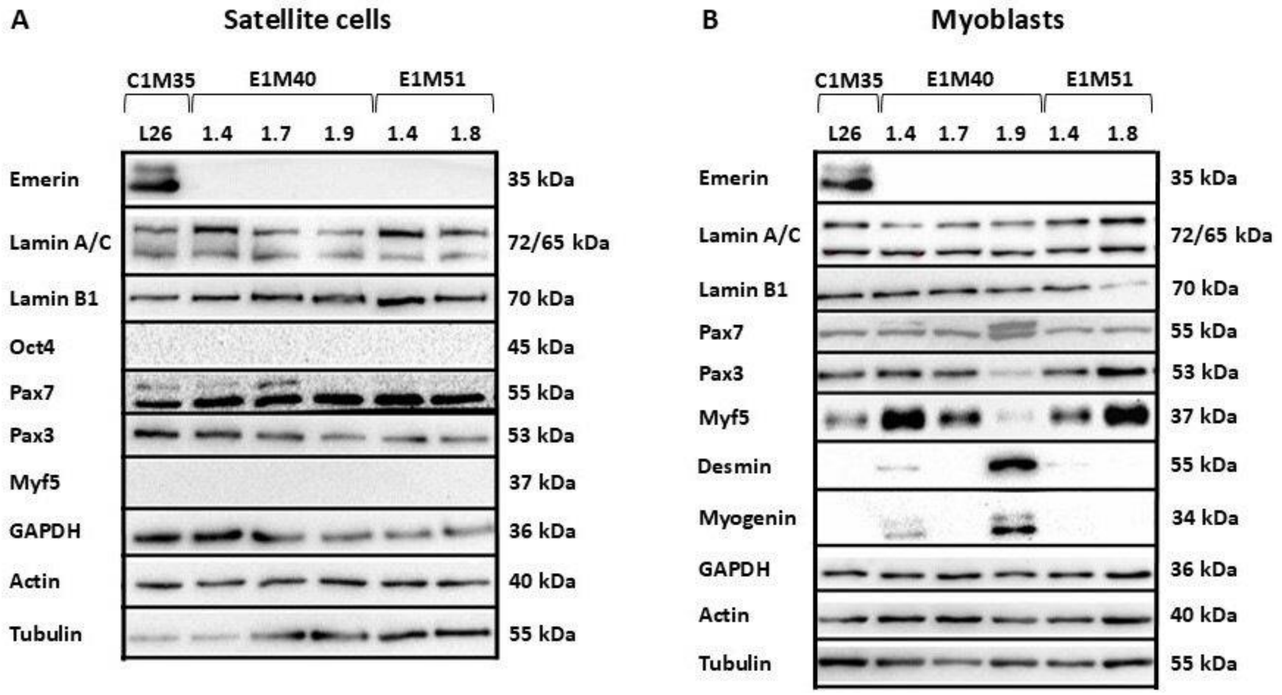

Figure S4. The analysis of protein levels in iPSC-derived SCs and myoblasts. One clone of iPSCs from a healthy donor (C1M35 L26) and five clones from two patients (E1M40 1.4, 1.7 and 1.9, and E1M51 1.4 and 1.8) with mutation c.153del (emerin null) were differentiated to SCs (A) and myoblasts (B). The samples were collected at different time points, once the cells reached 100% confluency, which occurred within 5-8 days for satellite cells and 4 to 6 days for myoblasts. Then, the expression of the following proteins was determined using western blot: nuclear-envelope proteins emerin, lamin A/C and lamin B; iPSCs marker Oct4; SCs markers Pax3 and Pax7; myoblasts marker Myf5; myotubes markers desmin and myogenin; and controls GAPDH, actin and tubulin. In both SCs and myoblasts, emerin is present only in the control line, while, as expected, lacking in the patient's cells (any faster-migrating form was not detected in optimized conditions; data not shown). In SCs, the expression of markers of particular stage of differentiation is proper - lack of iPSCs marker Oct4, expression of progenitor cells markers Pax3 and Pax7, and lack of Myf5 - marker of myoblasts - the subsequent stage of myogenesis. In myoblasts, Pax3 and Pax7 are still present, and the Myf5 is already expressed. In some clones, markers of myotubes (a subsequent stage of differentiation) - desmin and myogenin are present, which indicates that some cells started spontaneous differentiation.

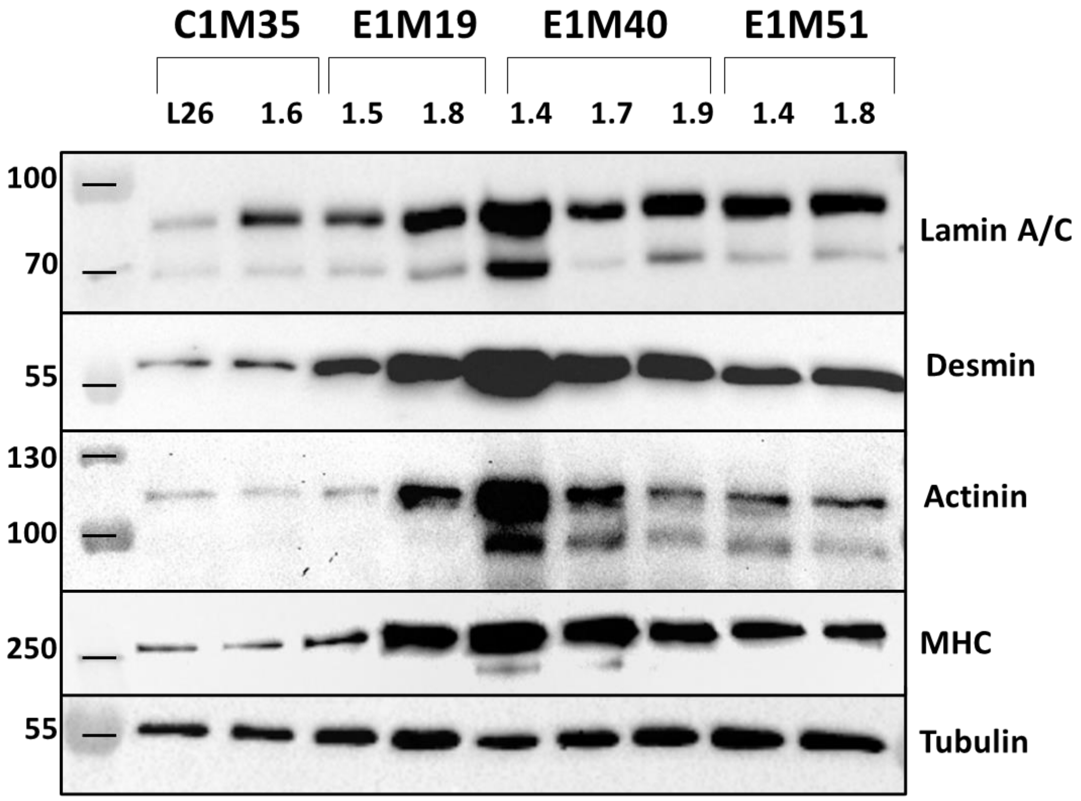

Figure S5. WB analysis of myogenesis markers in myotubes differentiated from iPSCs.
WB analysis was performed for two clones from healthy donor (C1M35) and seven clones from three patients. (E1M19, E1M40, E1M51). The presence of lamin A/C, desmin, MHC and actinin were confirmed in all clones, while proteins' levels depend on clone properties and differentiation time. Tubulin was used as loading control (the same staining as in the Figure 4C). Samples corresponding to WB presented on Figure 4C.
iPSC-derived myoblasts were cultured in myoblast medium for 1-3 weeks, depending on the clone, and differentiated to myotubes. C1M35 and E1M19 clones were able to differentiate into myotubes directly after medium change from myoblast medium to myotube medium, while E1M40 and E1M51 clones needed additional passaging and culturing in myoblast medium. WB present comparison of myotube samples obtained in the first possible time point for each clone.
